# Editorial: Non-cell Cycle Functions of Cell Cycle Regulators

**DOI:** 10.3389/fcell.2019.00122

**Published:** 2019-06-27

**Authors:** Song-Tao Liu, Gordon K. Chan, Weimin Li

**Affiliations:** ^1^Department of Biological Sciences, University of Toledo, Toledo, OH, United States; ^2^Department of Oncology, Faculty of Medicine and Dentistry, University of Alberta, Edmonton, AB, Canada; ^3^Department of Biomedical Sciences, Elson S. Floyd College of Medicine, Washington State University, Spokane, WA, United States

**Keywords:** cell cycle, p27Kip1, immune checkpoint, energy metabolism, insulin receptor (IR), mediator kinase, Cdks, mitotic checkpoint

In a canonical cell cycle of a proliferating mammalian cell, DNA is replicated during S phase and duplicated chromosomes are segregated into two daughter cells during mitosis (M) phase. Two gap phases, G1 and G2, precede S and M phases, respectively. The cell cycle is driven by various combinations of cyclins and CDKs, with most CDKs remaining constant at protein levels but cyclins oscillating through the cell cycle. CDKs require association with cyclins and phosphorylation or dephosphorylation to become active kinases. The accumulation of cyclins is usually driven by transcription while their degradation is mainly mediated by ubiquitination. The activities of cyclin-dependent CDKs are also influenced by CDK inhibitors, whose levels and subcellular localization are delicately controlled through transcriptional, translational and post-translational mechanisms. In addition, cell cycle checkpoints are imposed to regulate cyclins, CDKs, and CDK inhibitors. The checkpoints include restriction point in G1, DNA damage checkpoints including G1/S, intra S and G2/M checkpoints, and the spindle assembly checkpoint or the mitotic checkpoint during mitosis. Throughout the cell cycle, special macromolecular complexes such as the DNA replication origin recognition complexes, or specific subcellular structures such as the centromere-kinetochore complexes, are assembled to carry out spatiotemporally controlled functions.

Cell cycle regulators are commonly represented by cyclins, CDKs and CDK inhibitors, but can also include some of their substrates, interacting partners, and upstream regulators. These proteins have been well studied at molecular, cellular, and organismal levels in the context of cell proliferation control. However, additional functions have been uncovered for canonical cell cycle regulators, some of which have been discussed previously (Frank and Tsai, 2009; Lim and Kaldis, 2013; Hydbring et al., 2016). In the Research Topic “Non-cell cycle functions of cell cycle regulators,” several articles are presented to highlight exciting recent advances and provide more focused in-depth insights in several emerging areas.

There are 20 CDK and 29 cyclin genes in the human genome (Malumbres, 2014). Nine CDKs are not directly involved in cell cycle regulation but are engaged in transcriptional regulation. Dannappel et al. discussed two transcriptional CDKs, CDK8 and its paralog CDK19. These two kinases are termed “Mediator Kinase,” either of which, in a mutually exclusive manner, forms a complex with three other proteins: cyclin C, MED12, and MED13. MED12 and MED13 are two components of the Mediator complex required for RNA polymerase II-mediated transcription. The authors discussed the roles of CDK8 and CDK19 modules in signaling regulation in several organisms and in the context of development and diseases.

Coordination between metabolic fluxes, cell growth, and cell proliferation has gained more and more appreciation in recent years, partly due to molecular dissection of the metabolic alterations in cancer cells. Solaki and Ewald discussed how CDKs could target metabolic enzymes or their upstream regulators to control carbon and energy metabolism across different species. In some occasions, the metabolic re-wiring mediated by CDKs is to prepare for cell proliferation, but in many other cases the G1 CDKs and non-cell cycle CDKs regulate carbon and energy homeostasis even in differentiated post-mitotic cells. Our understandings on the roles of CDKs in energy metabolism are still in a nascent stage. For example, there are still controversies about whether and how mitochondrial respiration activities and intracellular ATP levels are regulated in a periodic fashion during the cell cycle.

Laphanuwat and Jirawatnotai discussed cell cycle regulators in immune modulation in both innate and adaptive immune systems. Although some aspects of the regulation might be intertwined with immune cell proliferation, others such as the regulation of T cell anergy by CDK2 and p27^kip1^ seem independent of their regular roles in cell cycle. The authors also reviewed recent development in overcoming tumor immune tolerance through the synergy between CDK4/6 small molecule inhibitors and immune checkpoint inhibitors.

Kawauchi and Nabeshima focused on the roles of cell cycle inhibitor p27^kip1^ in post-mitotic neurons, in particular its regulation of microfilament and microtubule cytoskeleton and possible involvement in membrane trafficking. They suggested that some core cell cycle regulators are re-purposed to gain extra-cell cycle regulatory functions (EXCERF) in post-mitotic cells while others are strictly suppressed in non-dividing cells. Accidental or forced expression of the latter class could cause cell death.

Finally, Choi and Yu discussed the context and implications of their recent surprising discovery that the spindle checkpoint proteins BUBR1, MAD2, and p31^comet^ regulate endocytosis of insulin receptor (Choi et al., 2016). It is interesting to note that their discovery satisfyingly explained the physiological importance of the MAD2-insulin receptor and BUBR1-Assembly Peptide 2 interactions reported about 20 years ago.

Although cell cycle regulators are traditionally studied at cellular levels, several articles in this series have pointed out that these regulators can reach beyond the cell-autonomous level to affect organismal health through impacting hormone or cytokine secretion and responses. Re-purposing of canonical cell cycle regulators for other functions in differentiated post-mitotic cells might be more widespread than currently realized. The intricate cell cycle regulatory network can be conveniently adapted for biological processes not directly related to cell proliferation, probably saving the effort to reinvent the wheels. The intrinsic disorder in some of the cell cycle regulatory proteins such as p27^kip1^ may form the physical foundation of their functional plasticity (Guharoy et al., 2015). Diverse post-translational modifications and sometimes mutations in the cell cycle regulators can also help expand their non-canonical interactions. It will be interesting to further study these re-purposing events under various physiological and pathological conditions.

We hope our readers enjoy the wonderful sampling of recent research on non-canonical functions of cell cycle regulators.

## Author Contributions

S-TL, GC, and WL prepared and finalized the manuscript based on a draft by S-TL.

### Conflict of Interest Statement

The authors declare that the research was conducted in the absence of any commercial or financial relationships that could be construed as a potential conflict of interest.
